# Alkyl Levulinates and 2-Methyltetrahydrofuran: Possible Biomass-Based Solvents in Palladium-Catalyzed Aminocarbonylation

**DOI:** 10.3390/molecules28010442

**Published:** 2023-01-03

**Authors:** Nuray Uzunlu, Péter Pongrácz, László Kollár, Attila Takács

**Affiliations:** 1 Department of General and Inorganic Chemistry, University of Pécs, Ifjúság Útja 6, H-7624 Pécs, Hungary; 2 János Szentágothai Research Centre, University of Pécs, Ifjúság Útja 20, H-7624 Pécs, Hungary; 3 ELKH-PTE Research Group for Selective Chemical Syntheses, Ifjúság Útja 6, H-7624 Pécs, Hungary

**Keywords:** aminocarbonylation, green solvents, amides, palladium, homogeneous catalysis

## Abstract

In this research, ethyl levulinate, methyl levulinate, and 2-methyltetrahydrofuran as bio-derived hemicellulose-based solvents were applied as green alternatives in palladium-catalyzed aminocarbonylation reactions. Iodobenzene and morpholine were used in optimization reactions under different conditions, such as temperatures, pressures, and ligands. It was shown that the XantPhos ligand had a great influence on conversion (98%) and chemoselectivity (100% carboxamide), compared with the monodentate PPh_3_. Following this study, the optimized conditions were used to extend the scope of substrates with nineteen candidates (various para-, ortho-, and meta-substituted iodobenzene derivatives and iodo-heteroarenes), as well as eight different amine nucleophiles.

## 1. Introduction

Biomass is the only abundant and concentrated source of non-fossil carbon that is available on Earth, and its conversion into special chemicals and fuels has been the focus of several chemical researches within the past decade [1]. The biomass obtained by plants and other wastes can be used to provide sustainable chemicals. In general, biomass usage will bring benefits, such as a cleaner environment, more security, and projected long-term economic savings [2]. The solvent industry, as one of the biggest and most important global markets, is projected to reach 30.0 billion USD in 2025 (21.8 billion USD in 2020) at a CAGR (compound annual growth rate) of 6.6% during the forecast period and is estimated to reach 34 million metric tons by 2027 [3,4].

The bio-derived solvents obtained from biomass are environmentally benign, biodegradable, and have lower toxicity than conventional organic solvents. Lomba and coworkers found in a detailed study that levulinic acid and its esters showed very low toxicity, with a high biodegrability, which supports their use as green alternatives of traditional chemicals. They investigated the ecotoxicity on *Chlamydomonas reinhardtii*, *Vibrio fischeri*, *Daphnia magna,* and *Eisenia foetida* and ascertained that the toxicities of the levulinates (methyl-, ethyl-, and butyl levulinate) were increased as a function of the length of the alkyl chain [5]. Ventura and her research group evaluated the toxicity of biomass-derived platform molecules by using the Microtox toxicity test. Contrary to the statement of the above-mentioned research group, they found that ethyl levulinate was less toxic (practically harmless, EC_50_ = 694 mg L^−1^) than levulinic acid (moderately toxic, EC_50_ = 28.4 mg L^−1^) in the case of *Vibrio fischeri* bacteria [6]. In recent times, 2-MeTHF has been selected over tetrahydrofurane, due to its favorable characteristic properties (e.g., higher boiling point, which allows performing reactions at a relatively higher temperature [7], lower miscibility in water, which favors a cleaner workup [8]). Furthermore, it has been established after detailed toxicological studies that 2-MeTHF is not associated with any genotoxicity and mutagenicity [9].

Because solvents have great influence on reactivity and selectivity, their selection is very crucial in most chemical processes. For this reason, there are solvent selection guides made by researchers in academic and industrial areas (e.g., CHEM21, Sanofi’s selection guide) to help choose the appropriate reaction media for a chemical synthesis [10,11,12,13]. In these guides, the preferable terms are characterized by favorable environmental, health, and safety (EHS) properties [14], and chemicals are categorized generally as hazardous, problematic, or recommended. On the other hand, these guides are helpful for selecting the best solvent because the “green solvents” deal with constraints that are sometimes contradictory: the chemical reaction efficiency, safety (flash point, resistance, energy decomposition, and peroxydation), health (acute, long-term, and single-target organ toxicity), environment (biodegradability, ecotoxicity, solubility in water, volatility, odor, and life cycle analysis), quality, industrial constraints (boiling and freezing points, density, and recyclability) and cost. Briefly, the best green solvent must meet the specifications by which the concept of “green chemistry” has been defined by the well-known 12 principles [15].

Water is cheap, safe, non-toxic, environmentally benign, and readily available; therefore, the application of it as a suitable reaction medium has received increasing attention during the last decades. However, an efficient homogeneous CC coupling, as well as an aminocarbonylation reaction, are often restricted, due to the limited solubility of reagents (substrates, amine nucleophiles) and amide products or the decomposition of the catalyst. Consequently, many more heterogeneous [16,17,18,19,20,21] or biphasic [22] aminocarbonylation processes have been described than homogeneous processes [23,24,25]. Additionally, it has been described by our research group that water can act as an *O*-nucleophile during aminocarbonylative conditions, resulting in the formation of carboxylic acid when low-reactive nucleophile reaction partners are used [26]. Considering the above-mentioned reasons, our attention turned to using bio-derived solvents instead of water.

Transition metal-catalyzed carbonylation reactions in the presence of nucleophile reagents have become an indispensable tool for the synthesis of several α,β-unsaturated and aromatic carboxylic acid derivatives [27,28]. Although these reactions require low catalyst loading, they generally need a huge amount of solvent as a reaction media. One of these reactions is aminocarbonylation, which has great importance concerning the synthesis of simple building blocks and the functionalization of biologically important skeletons. Carboxamides can be synthesized from easily available starting materials with aminocarbonylation, although the conventional carboxylic acid−carboxylic halide−carboxamide route is difficult to prepare because it has no notable yield in the implementation [29,30]. Moreover, our research group has been investigating this reaction in the conventional solvent DMF for years [29,30,31,32,33,34,35,36,37,38,39], and thanks to the ascertainments of the groups from Skrydstrup [40] and Mika [41,42,43], our interest has been turned to the investigation of green solvents.

In this study, we plan to investigate some appropriate green solvents as reaction mediums for Pd-catalyzed aminocarbonylation, which has many applications in both industrial and fine chemistry in organic synthesis, including the introduction of amides with a variety of *N*-substituents [44,45]. Amides are one of the most important classes of organic compounds, especially for the pharmaceutical industry, because most of the drugs contain amide functionality. For example, most of the top 15 best-selling drugs in 2017 contained amide moiety [46].

**Figure 1:**
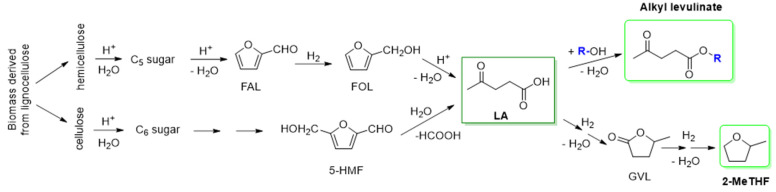
Converting lignocellulose through levulinic acid to alkyl levulinates and 2-MeTHF [52,53].

Levulinic acid (LA), which is one of the platform compounds derived from biomass, can be produced from lignocellulose biomass via two different ways. One is the direct hydrolysis of biomass based on cellulose through 5-(hydroxymethyl)furfural (5-HMF) intermediate, while the other is known as the furfuralcohol catalytic hydrolysis way, producing LA via the hemicellulose–xylose(C_5_ unit)–furfural–furfuralcohol pathway (Figure 1). Therefore, levulinic acid can be considered a valuable platform molecule that can be converted into several important chemicals, such as levulinate esters, GVL, 2-MeTHF, etc. In this work, ethyl levulinate was chosen as a biomass-derived solvent that can be synthesized via direct esterification of the platform molecule, levulinic acid, in ethanol (Figure 1). This bio-based solvent is the viable additive for gasoline and diesel transportation fuels, and it can also either be used in the flavoring and fragrance industries or as substrates for various kinds of condensation and addition reactions at the ester and keto groups in organic chemistry [47,48]. Lei et al. investigated the Suzuki−Miyaura coupling of amides with this solvent and obtained 63% conversion during this transformation [49]. Another chosen levulinate ester was methyl levulinate, which is also a certified viable additive for gasoline and diesel transportation fuels, similar to the other levulinate esters [50]. Homogeneous acid catalysts or mixtures of Lewis and Brønsted acids have also been generally employed to produce a high yield of methyl levulinate from cellulose [51].

According to the computational study of Leal Silva’s group, the conversion of furfural derived from hemicellulose to 2-methyltetrahydrofuran (2-MeTHF) could be more profitable than ethyl levulinate synthesis [52], which moved our attention to the advantages of 2-MeTHF as a solvent in the aminocarbonylation reaction. It is highly flammable and mostly used as a fuel additive and an alternative solvent of tetrahydrofuran (THF) [47,54]. 2-MeTHF is a promising solvent for transition metal-catalyzed reactions, and it has been justified with quite a large number of articles published in literature [28,54,55,56,57,58,59,60,61,62,63]. In this research, considering the green properties of the above-mentioned alkyl levulinates and 2-MeTHF, we investigated their applicability in the palladium-catalyzed aminocarbonylation of iodobenzene and its substituted derivatives, as well as iodo(hetero)arenes, in the presence of various *N*-nucleophiles.

## 2. Results and Discussion

### 2.1. Optimization Study

**Scheme 1:**
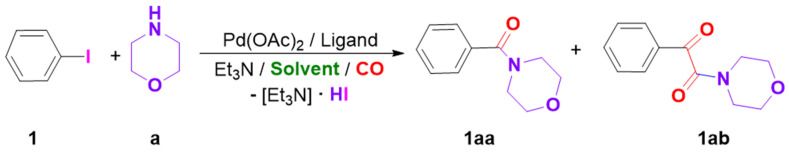
Palladium-catalyzed aminocarbonylation reaction of iodobenzene with morpholine.

**Figure 2:**
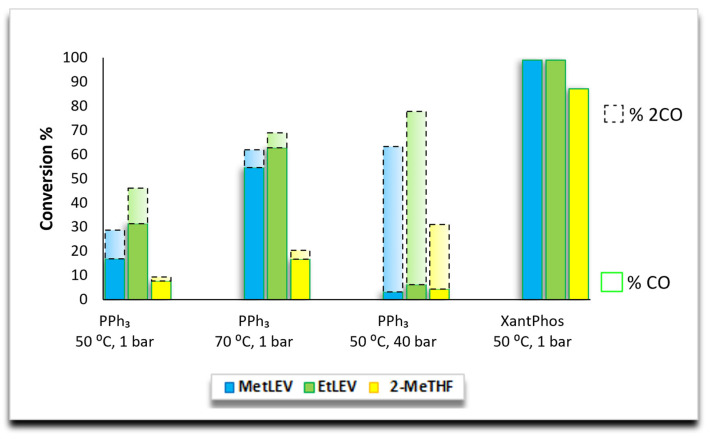
Optimization study of the aminocarbonylation of iodobenzene (**1**) with morpholine (**a**). (Reaction conditions: 0.5 mmol of iodobenzene, 0.75 mmol of morpholine, 0.0125 mmol of Pd(OAc)_2_, 0.025 mmol of PPh_3_ or 0.0125 mmol of XantPhos, 0.25 mL of triethylamine, and 5 mL of solvent under a CO atmosphere for 6 h. The conversion was determined by GC using dodecane as an internal standard.)

Iodobenzene and morpholine as the nucleophilic reaction partners were chosen to find the optimized conditions in our aminocarbonylation model reaction performed in green solvents (Scheme 1). Pressure, temperature, and ligand were selected as the variable parameters in the optimization study (Figure 2). First, the reaction was performed in the presence of a Pd(OAc)_2_/2 PPh_3_ catalyst at 50 °C under 1 or 40 bar CO, conditions which have been generally used and well-studied by our research group [64].

Under atmospheric carbon monoxide pressure, the reaction showed moderate conversion (29–46%) in the alkyl levulinate-type solvents (MetLEV and EtLEV) and 9% conversion in 2-MeTHF after 6 h. Under these conditions, the reaction was not chemoselective, due to the formation of the corresponding 2-ketocarboxamide-type product (16–41%) caused by the double carbon monoxide insertion. After carrying out the reactions under elevated temperature (70 °C) in the presence of the previously used catalyst, the following considerations are worth mentioning: (i) the chemoselectivity was favorable toward the amide (**1aa**) product, and (ii) the conversions were increased in the case of all solvents after a 6 h reaction time. The CO pressure had a great influence on the product selectivity. After carrying out the reaction at 40 bar of carbon monoxide pressure in the presence of the previously used catalyst at 50 °C, the 1-morpholino-2-phenylethane-1,2-dione (**1ab**) formed with high chemoselectivity (>85%) in all cases. It must be noted that the iodobenzene (**1**) was converted by 79% by using the alkyl levulinate-type solvents, while in 2-MeTHF, the conversion was only 35% after a 24 h reaction. Despite the promising chemoselectivity results at the elevated CO pressure, the above-mentioned low conversions inspired us to find other reaction conditions that could increase the synthetic importance of this reaction in green solvents. The aminocarbonylation of iodobenzene and *tert*-butylamine has been investigated by Marosvölgyi-Haskó and coworkers in DMF [65] and GVL using the Pd(OAc)_2_/PPh_3_ catalyst [41]. Although the amines were different, we described similar behavior in our former research (reactivity and selectivity) under the same reaction conditions [66,67,68]. After carrying out the reactions with *tert*-butylamine in the conventional organic solvent, the following considerations can be stated after a 6 h reaction time: *(i)* the conversion (50%) was higher under atmospheric conditions than in our green solvents (9–46%); *(ii)* the chemoselectivity was better towards the ketoamide-type product in DMF (amide:ketoamide = 22:78) at 1 bar of CO, while the amide formation was more favorable in alkyl levulinates and 2-MeTHF (59–84%); *(iii)* under elevated carbon monoxide pressure, higher conversions were detected in alkyl levulinates (63–78%) than in DMF (53%); *(iv)* excellent chemoselectivity towards the ketoamide was observed in the conventional solvents, as well as in our green solvents. It also has to be mentioned that the conversions and the chemoselectivity values, observed in GVL by Marosvölgyi-Haskó et. al., are similar to our results detected in alkyl levulinates.

Based on our former research [69,70], in which XantPhos was used successfully to convert the substrate selectively to the target product in a short reaction time, we decided to apply this rigid bidentate phosphine ligand with a large bite angle to increase the efficiency (e.g., conversion, selectivity) of the model reaction. After changing the triphenylphosphine to XantPhos and carrying out the reaction under ambient conditions (50 °C and 1 bar of CO pressure), complete conversion was detected in the alkyl levulinate solvents, while the reaction was also almost complete in 2-MeTHF (87%) after 6 h.

To justify the applicability of the bio-derived candidates, we performed our model reaction in DMF under the same reaction conditions as in the green solvents, and the results are summarized in Table 1.

It can be seen that the conversion in DMF was higher than in our solvents under atmospheric conditions and in the presence of the Pd(OAc)_2_/2 PPh_3_ catalyst system. Furthermore, the formation of the **1ab** product was more favorable in DMF, while the amide (**1aa**) formation was preferable in the green solvents (entries 1, 5, 9, and 13). The amide (**1aa**) formation was much more expressed by carrying out the reactions at 70 °C (entries 2, 6, 10, and 14). The conversion and the selectivity values were almost the same in conventional and green solvents (except in 2-MeTHF) under high pressure conditions (entries 3, 7, 11, and 15). After changing the triphenylphosphine to the bidentate XantPhos, the reaction rate was extremely increased, and the amide (**1aa**) was formed with high selectivity (88%) (entries 4, 8, 12, and 16).

Additionally, the morpholino(phenyl)methanone (**1aa**) compounds were isolated in the following yields after performing the aminocarbonylation under optimized conditions: 61% (MetLev), 66% (EtLev), and 65% (2-MeTHF). After comparing these values with the isolated yields (>81%) reached by using conventional solvents [71,72,73], it can be stated that removing the solvent under reduced pressure followed by column chromatography is an applicable process to isolate the carboxamide products synthesized in bio-derived solvents. In this way, we have appropriate conditions to use in further reactions, in which we can extend the scope of the amine nucleophiles, as well as the substrates.

### 2.2. Extending the Scope of Amine Nucleophiles

**Figure 3:**
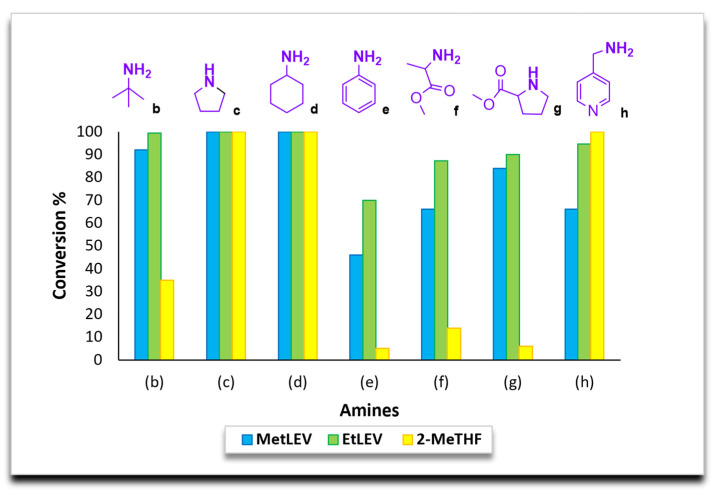
Palladium-catalyzed aminocarbonylation of iodobenzene (**1**) with different amines (**b**–**h**). (Reaction conditions: 0.5 mmol of iodobenzene, amine nucleophile (1.5 mmol of tert-butylamine, 0.75 mmol of pyrrolidine, 0.75 mmol of cyclohexylamine, 1.0 mmol of aniline, 0.55 mmol of amino acid methyl esters (AlaOMe, ProOMe), and 0.75 mmol of 4-picolylamine), 0.0125 mmol of Pd(OAc)_2_, 0.0125 mmol of XantPhos, 0.25 mL of triethylamine, and 5 mL of solvent at 50 °C under 1 bar of CO in 6 h. The conversion was determined by GC using dodecane as an internal standard.)

With the optimized conditions on hand (XantPhos, 50 °C, atmospheric carbon monoxide pressure), we extended the scope of the amine nucleophiles in the aminocarbonylation of iodobenzene (Figure 3).

It can be seen, by using simple primary (**b**,**d**) and secondary amines, that they showed complete conversion in all green solvents. The tert-butylamine (**b**) provided similar reactivity to amine (**c**) and (**d**) alkyl levulinate solvents, while in 2-MeTHF, strikingly lower conversion was detected after 6 h. The lowest basicity among our nucleophiles was in the presence of the aromatic aniline (**e**), with 46 and 70% conversions detected in methyl and ethyl levulinate, respectively. The N-phenylbenzamide (**2ea**) was identified in traces in 2-MeTHF. Alanine methyl ester (**f**) and proline methyl ester (**g**) showed a slightly lower reactivity in methyl levulinate than in ethyl levulinate, but the difference was not significant. Furthermore, the two amino acid methyl esters provided the lowest conversion in 2-MeTHF, which can be explained by their low solubility in this solvent. While the 4-picolylamine (**h**) showed similar reactivity to the amino acid methyl esters in alkyl levulinates, surprisingly, it was completely converted to the corresponding carboxamide (**2ha**) in 2-MeTHF after a 6 h reaction.

### 2.3. Extending the Scope of Substrates

**Scheme 2:**
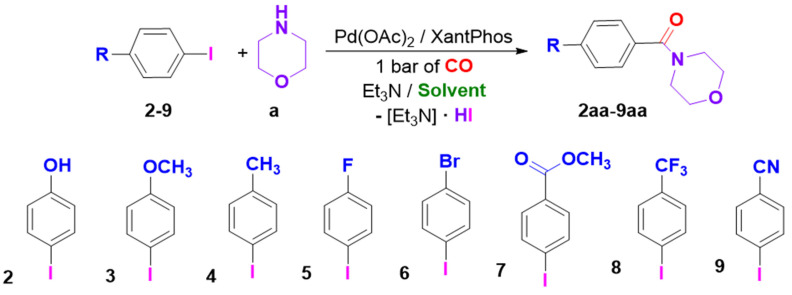
Palladium-catalyzed aminocarbonylation reaction with para-substituted iodobenzenes (**2**–**9**) and morpholine (**a**).

In the next step, iodobenzene derivatives (**2**–**9**) possessing various para-substituents were reacted with morpholine (a) in the chosen green solvents under carbonylative conditions (Scheme 2). 

**Figure 4:**
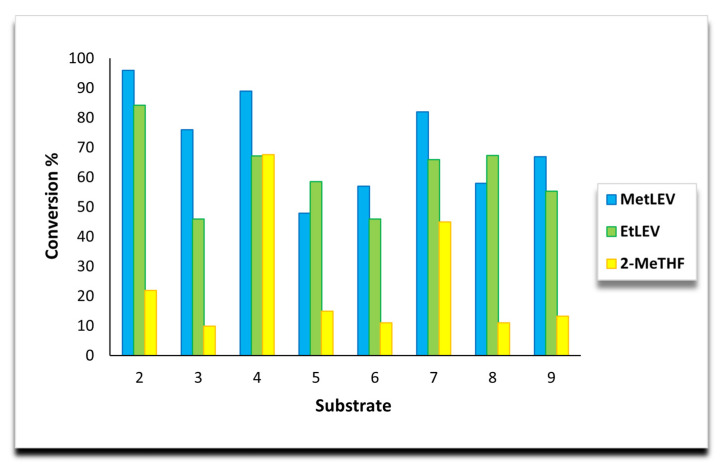
Palladium-catalyzed aminocarbonylation of para-substituted iodobenzene derivatives (4-iodo-phenole, 4-iodoanisole, 4-iodotoluene, 1-fluoro-4-iodobenzene, 1-bromo-4-iodobenzene, methyl 4-iodo-benzoate, 4-iodobenzotrifluoride, and 4-iodobenzonitrile). (Reaction conditions: 0.5 mmol of para-substituted iodobenzene, 0.75 mmol of morpholine, 0.0125 mmol of Pd(OAc)_2_, 0.0125 mmol of XantPhos, 0.25 mL of triethylamine, and 5 mL of solvent under a CO atmosphere for 2 h. The conversion was determined by GC using dodecane as an internal standard.)

Although a strong relation between the reactivity and the Hammett para constant of the substrates was not observed, the substrates (**8**, **9**), having electron-withdrawing substituents, provided lower conversions (55–67%) than the first three para-substituted iodobenzene derivatives (**2**–**4**) that bore electron-donating groups (Figure 4). Furthermore, substrates **2**–**4** reacted quite quickly with morpholine and gave high conversion values (76–96%), especially in methyl levulinate, after a 2 h reaction. It can also be seen that 1-fluoro-4-iodobenzene (**5**) and 1-bromo-4-iodobenzene (**6**) showed the lowest reactivity (46–59% conversions) in the alkyl levulinate solvents. After analyzing the reaction mixtures after 6 h, complete conversions were detected in almost all cases except 4-iodoanisole **3** (92%). Each substrate showed quite a high reactivity in alkyl levulinate-type solvents (>46%) in a 2 h reaction. It can also be seen that the behaviors of the para-substituted model compounds are almost completely the same. Only substrate **3** provided a much lower reactivity in the EtLEV (46%) than in the MetLEV (76%/) solvent. Consequently, there was no significant difference between the behaviors of our para-substituted substrates (**2**–**9**) in the alkyl levulinate-type solvents. The reactions, performed in 2-MeTHF under the same conditions mentioned above, showed strikingly different reactivity than those in the alkyl levulinate solvents. Most of the substrates showed very low conversions (<22%) in 2 h, except 4-iodotoluene (**4**) and methyl 4-iodobenzoate (**7**), in which cases 68% and 45% conversions were observed, respectively. While substrates **4** and **7** provided complete conversion, the others (**2**, **3**, **5**, **6**, **8**, and **9**) were converted by only 24–68% after a 6 h reaction. More than 92% of the target compounds were detected in the reaction mixture after 24 h; only the 4-iodobenzotrifluoride (**8**) provided the corresponding carboxamide in a quantity of 78%.

**Figure 5:**
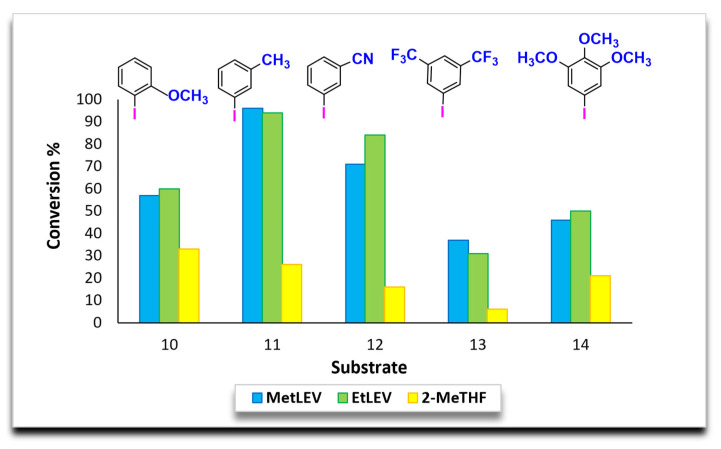
Pd-catalyzed aminocarbonylation of substituted iodobenzenes (**10**–**14**) with morpholine (**a**). (Reaction conditions: 0.5 mmol of substrate, 0.75 mmol of morpholine, 0.0125 mmol of Pd(OAc)_2_, 0.0125 mmol of XantPhos, 0.25 mL of triethylamine, and 5 mL of solvent under CO atmosphere in 6 h. The conversion was determined by GC using dodecane as an internal standard.)

In the continuation, ortho/meta-mono-substituted (**10**–**12**) and di- and tri-substituted (**13**, **14**) iodobenzene derivatives were examined. Compared to para-substituted substrates, the ortho-, di-, and tri-substituted compounds (**10**, **13**, **14**) provided lower conversions, likely due to steric reasons (Figure 5).

The 3-iodotoluene (**11**) (96 and 94% conversion) and 3-iodobenzonitrile (**12**) (71 and 84% conversion) showed high activity in both MetLEV and EtLEV solvents after 6 h reactions, while 2-iodoanisole (**10**) was converted in moderate yields (57–60%), due to the steric hindrance of the methoxy moiety in the ortho position. Substrate **13**, which had trifluoromethyl substituents in ortho- and para-positions, showed less reactivity (32–37%) in alkyl levulinate solvents, while the tri-substituted 5-iodo-1,2,3-trimethoxybenzene (**14**) was slightly more reactive than 1-iodo-3,5-bis(trifluoromethyl)benzene (**13)** during the 6 h reaction. Furthermore, it was demonstrated that substrates **11**, **12**, and **14** were converted completely, in contrast with substrates **10** and **13**, which gave 80–93% conversions in alkyl levulinates after a 24 h reaction. Considering our result in the aminocarbonylation reaction with substrates **11**–**14** and morpholine in 2-MeTHF, it can be easily seen that each starting material showed very poor reactivity (<33%) after 6 h. Nonetheless, substrate **11** had a complete conversion in 24 h, while substrates **10**, **12**, and **14** were converted by 58–65% in one-day reactions. Only substrate **13** was converted slowly, resulting in the (2,4 bis(trifluoromethyl)phenyl) (morpholino)methanone (**13aa**) product being detectable in traces (16%) after 24 h. Consequently, we can underline that alkyl levulinate-type solvents could be more appropriate solvents than 2-MeTHF in the aminocarbonylation reaction in the presence of ortho/meta-mono-substituted (**10**–**12**) and di/tri-substituted (**13**, **14**) iodobenzene derivatives.

**Figure 6:**
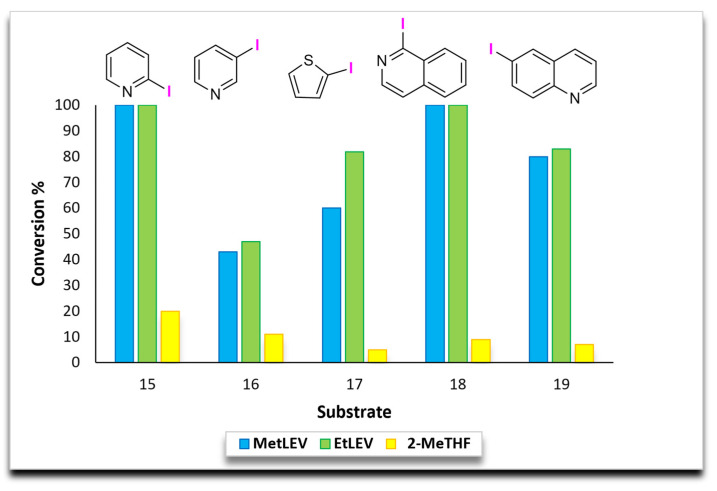
Pd-catalyzed aminocarbonylation of iodoheteroaromatic substrates (**15**–**19**) with morpholine (**a**). (Reaction conditions: 0.5 mmol of substrate, 0.75 mmol of morpholine, 0.0125 mmol of Pd(OAc)_2_, 0.0125 mmol of XantPhos, 0.25 mL of triethylamine, and 5 mL of solvent under CO atmosphere in 2 h. The conversion was determined by GC using dodecane as an internal standard.)

In the last part of the study, some iodo-heteroaromatic substrates (**15**–**19**) were reacted with morpholine under optimized conditions. Then, their reactivities were investigated and compared in the green solvents above (Figure 6). 

2-Iodopyridine (**15**) and 1-iodoisoquinoline (**18**), in which the iodo-functionality is adjacent to the N-atom in the ring, were completely converted into the target carboxamide (**15aa**, **18aa**) in methyl- and ethyl levulinate after a 2 h reaction. 2-Iodothiophene (**17**) and 6-iodoquinoline (**19**) also showed good conversions (60–83%), while 3-iodopyridine (**16**) was the least reactive (43–47%) among them. While substrates **17** and **19** were converted completely in 6 h, 3-iodopyridine (**16**) showed 88% and 92% conversions in MetLEV and EtLEV, respectively. It is clearly seen in Figure 6 that the substrates unambiguously showed much higher reactivity in the alkyl levulinate-type solvents than in 2-MeTHF after 2 h of reaction time; conversion values lower than 20% were detected by GC analysis. The most reactive 2-iodopyridine (**15)** provided the target morpholino(pyridin-2-yl)methanone (**15aa**) compound with 82% conversion while in the reaction mixture, even though the conversions were just 7–36%, in the case of substrates **16**–**19**, after 6 h in 2-MeTHF.

**Scheme 3:**
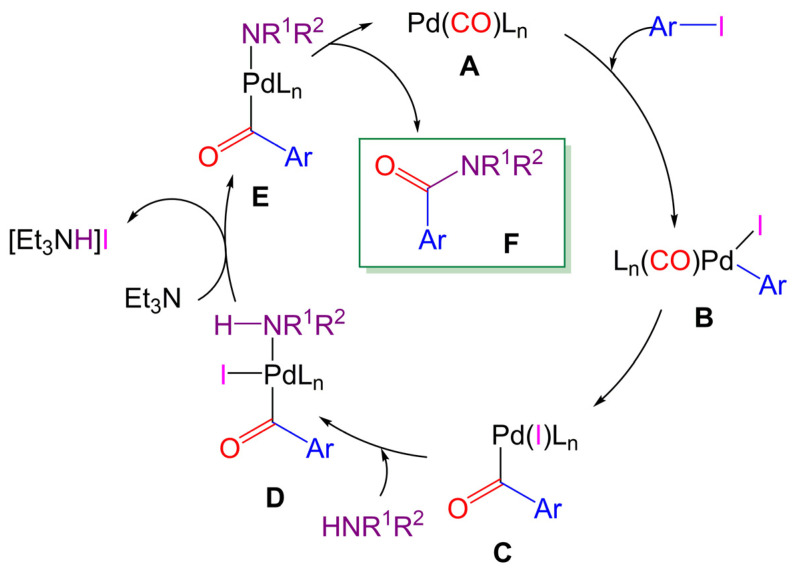
Proposed mechanism of palladium-catalyzed aminocarbonylation in bio-derived solvents.

Because the conversions, especially in alkyl levulinates, are comparable to the reactions performed in conventional solvents, and the isolated yields in the case of compound **1aa** are similar to the results found in literature, the elementary steps of the catalytic cycle should be the same as in conventional solvents. Considering this concept, the following mechanism could be proposed. The ‘starting’ palladium(0) complex (Pd(CO)L_n_, **A**) formed by reduction, reacts with the organic halide substrate in an oxidative addition, affording the palladium(II)-aryl intermediate (**B**). It is followed by the insertion of carbon monoxide into the palladium–carbon (Pd-Ar) bond, resulting in the corresponding palladium(II)-acyl complex (**C**). The next step is the nucleophile (NuH) attack on the species **C,** giving the catalytic intermediate **D**. It is followed by the HI elimination in the presence of the base (Et_3_N) from the complex **D,** providing the amido-acyl-palladium(II) species (**E**). The last step is the reductive elimination, in which the carboxamide (**F**) is formed, and the palladium(0) species is regenerated (Scheme 3).

## 3. Materials and Methods

### 3.1. Compounds and Solvents

Solvents (ethyl levulinate, methyl levulinate, 2-MeTHF, and dichloromethane), substrates (**1**–**19**), nucleophiles (**a**–**h**), and triethylamine (Et_3_N) were purchased from Sigma-Aldrich (St. Louis, MO, USA), and they were used without any further purification. The Pd(OAc)_2_ and the ligands (PPh_3_ and XantPhos) were also purchased from Sigma-Aldrich. TLC plates (silica gel on TLC Al foils with fluorescence indicators of 254 nm) and the silica gel (high-purity grade, average pore size 60 Å (52–73 Å), 70–230 mesh) were purchased from Sigma-Aldrich (St. Louis, MO, USA).

### 3.2. Aminocarbonylation Reaction under Atmospheric Pressure of CO

Pd(OAc)_2_ (2.8 mg, 0.0125 mmol), PPh_3_ (6.5 mg, 0.025 mmol), or XantPhos (7.2 mg, 0.0125 mmol) were measured in a 100 mL three-necked flask equipped with a reflux condenser connected to a balloon filled with argon. An amount of 0.5 mmol of substrate, one of the amine nucleophiles (0.75 mmol of 4-picolylamine, cyclohexylamine, pyrrolidine, and morpholine; 0.55 mmol of ProOMe∙HCl and AlaOMe∙HCl; 1.0 mmol of aniline; and 1.5 mmol of ^t^BuNH_2_), 0.25 mL of triethylamine, 0.25 mmol of internal standard (dodecane), and 5 mL of the green solvent were added under argon. Then the balloon was vacuumed and filled with carbon monoxide. The reaction mixture was stirred at 600 rpm in an oil bath at 50 °C for 24 h. The conversion was determined by GC measurements after 2, 6, and 24 h, and then the reaction was stopped and filtered. All the carboxamides, synthesized in the aminocarbonylation reactions, have already been described and characterized in previous literature. Due to this reason and environmental considerations, the isolation of the products was not performed. We performed the workup and isolation processes only in the case of our model aminocarbonylation reaction, which was performed in the presence of XantPhos; the solvents were removed under reduced pressure, and the crud was purified using a column chromatography with the carefully chosen CHCl_3_: EtOAc = 8:2 eluent.

### 3.3. Aminocarbonylation Reaction under High Pressure of CO

Pd(OAc)_2_ (2.8 mg, 0.0125 mmol), PPh_3_ (6.5 mg, 0.025 mmol), or XantPhos (7.2 mg, 0.0125 mmol) were measured into a 100 mL stainless steel autoclave. The reagents and solvents, which were mentioned in Chapter 2.2, were transferred under argon. The reaction vessel was pressurized up to 40 bar total pressure with carbon monoxide, and the reaction mixture was stirred at 600 rpm in an oil bath at 50 °C for 24 h. After the given time, the reaction was stopped, and the autoclave was carefully depressurized in a well-ventilated hood. Then, the reaction mixture was filtered and analyzed by GC measurements after 2, 6, and 24 h.

### 3.4. Analytical Measurements

The reaction mixtures were analyzed by gas chromatography (Shimadzu Nexis GC-2030, Tokyo, Japan; Agilent J&W GC Column, DB-1MS stationary phase with automatic injection or DB-5MS stationary phase for ethyl levulinate and 1-iodobenzene with manual injection) using the following parameters: injector temperature: 250 °C; oven initial temperature: 50 °C (holding time: 1 min.); heating rate: 15 °C/min; final temperature: 320 °C (holding time: 11 min.); detector temperature: 280 °C; and carrier gas: helium (1 mL/min). The conversion and selectivity of the reactions were determined by GC. Unless otherwise stated, the conversion was checked with the internal standard method using dodecane. Mass spectrometry data were recorded using a GC–MS-QP2020 system (Shimadzu, Tokyo, Japan) with electron spray ionization (ESI) to identify the amides formed in the reactions (See Supplementary materials). The data are given as mass unit per charge (m/z), and the intensities are given in brackets. These data sets can be found in the Supplementary file.

## 4. Conclusions

In conclusion, we focused our attention on selecting environmentally friendly and greener solvents, which are commercially available and quite new, to facilitate the development of palladium-catalyzed homogeneous aminocarbonylation reactions. After the detailed optimization study, it has been shown that the Pd(OAc)_2_/XantPhos catalyst system has great activity in the aminocarbonylation reaction of iodobenzene and morpholine, and the substrate converted completely and selectively towards the target carboxamide. By performing our model reaction in a conventional solvent, we demonstrated that the results observed in DMF and in the bio-derived reaction media are similar, justifying the applicability of our green solvents in aminocarbonylation. The appropriate conditions were chosen to extend the scope of amine nucleophiles with eight candidates, as well as nineteen different substrates (various *para*-, *ortho*-, and *meta*-substituted iodobenzene derivatives and iodo-heteroarenes). Considering our results, it can be unambiguously stated that the methyl- and ethyl levulinate are much more effective than the 2-MeTHF under similar conditions. Consequently, the ’alkyl levulinate’-type solvents could be used as alternative solvents for palladium-catalyzed aminocarbonylation reactions, opening a greener procedure for this synthetically relevant transformation.

## Figures and Tables

**Table 1:** Comparison of the aminocarbonylation reaction of iodobenzene (**1**) and morpholine (**a**) in conventional and green solvents ^(a)^.

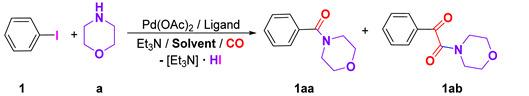							
Entry	Solvent	Ligand	Temp.	p_co_	Conv.^(b)^	Ratio of Products ^(b)^	
			[°C]	[bar]	[%]	Amide	Ketoamide
1	DMF	PPh₃	50	1	71	28	72
2	DMF	PPh₃	70	1	74	67	33
3	DMF	PPh₃	50	40	79	3	97
4	DMF	XantPhos	50	1	100	88	12
5	MetLev	PPh₃	50	1	29	59	41
6	MetLev	PPh₃	70	1	62	88	12
7	MetLev	PPh₃	50	40	63	5	95
8	MetLev	XantPhos	50	1	99	100	0
9	EtLev	PPh₃	50	1	46	68	32
10	EtLev	PPh₃	70	1	69	91	9
11	EtLev	PPh₃	50	40	78	8	92
12	EtLev	XantPhos	50	1	98	100	0
13	2-MeTHF	PPh₃	50	1	9	84	16
14	2-MeTHF	PPh₃	70	1	20	82	18
15	2-MeTHF	PPh₃	50	40	31	14	86
16	2-MeTHF	XantPhos	50	1	87	100	0

^(a)^ Reaction conditions: 0.5 mmol of iodobenzene, 0.75 mmol of morpholine, 0.0125 mmol of Pd(OAc)_2_, 0.025 mmol of PPh_3_ or 0.0125 mmol of XantPhos, 0.25 mL of triethylamine, and 5 mL of solvent under a CO atmosphere after a 6 h reaction time. ^(b)^ The conversion and the ratio of the carbonylated products were determined by GC using dodecane as an internal standard.

## Data Availability

The data presented in this study are available in the Supplementary Material.

## Supplementary Materials

The following supporting information can be downloaded at: https://www.mdpi.com/article/10.3390/molecules28010442/s1, Table S1: Optimization study of the aminocarbonylation of iodobenzene (1) with morpholine (a); Table S2: Palladium-catalyzed aminocarbonylation of iodobenzene (1) with different primary and secondary amines (b–h); Table S3: Palladium-catalyzed aminocarbonylation of para-substituted iodobenzene derivatives (**2**–**9**) with morpholine (a); Table S4: Palladium-catalyzed aminocarbonylation of substituted iodobenzenes (**10**–**14**) with morpholine (a); Table S5: Palladium-catalyzed aminocarbonylation of iodoheteroaromatic substrates (**15**–**19**) with morpholine (a); Table S6: Palladium-catalyzed aminocarbonylation of iodobenzene (1) with different primary and secondary amines (a–h); Table S7: Palladium-catalyzed aminocarbonylation of para-substituted iodobenzene derivatives (**2**–**9**) with morpholine (a); Table S8: Palladium-catalyzed aminocarbonylation of substituted iodobenzenes (**10**–**14**) with morpholine (a); Table S9: Palladium-catalyzed aminocarbonylation of iodoheteroaromatic substrates (**15**–**19**) with morpholine (a).

## Sample Availability

Samples of the compounds are not available from the authors.
