# Supplementary material for: Alkyl Levulinates and 2-Methyltetrahydrofuran: Possible Biomass-Based Solvents in Palladium-Catalyzed Aminocarbonylation
Source: Molecules. 2023 Jan 3;28(1):442. doi: 10.3390/molecules28010442 (PMC9823927; doi:10.3390/molecules28010442)
Supplement: Supplementary file 1 [file molecules-28-00442-s001.zip › molecules-2052857-supplementary.pdf]

Supporting Information

Alkyl Levulinates and 2-Methyltetrahydrofuran: Possible Biomass-Based Solvents in Palladium-Catalyzed Aminocarbonylation

Nuray Uzunlu [1], Péter Pongrácz [1], László Kollár [1, 2, 3] and Attila Takács [1, 2,*]

[1] *Department of General and Inorganic Chemistry, University of Pécs, Ifjúság útja 6., H-7624 Pécs, Hungary*
[2] *János Szentágothai Research Centre, University of Pécs, Ifjúság útja 20., H-7624 Pécs, Hungary*
[3] *ELKH-PTE Research Group for Selective Chemical Syntheses, Ifjúság útja 6., H-7624 Pécs, Hungary*
Correspondence: takacsattila@gamma.ttk.pte.hu

Contents

I. Conversions determined by GC

Table S1: Optimization study of the aminocarbonylation of iodobenzene (**1**) with morpholine (**a**).

Table S2: Palladium-catalyzed aminocarbonylation of iodobenzene (**1**) with different primary and secondary amines (**b**-**h**).

Table S3: Palladium-catalyzed aminocarbonylation of *para*-substituted iodobenzene derivatives (**2-9**) with morpholine (**a**).

Table S4: Palladium-catalyzed aminocarbonylation of substituted iodobenzenes (**10-14**) with morpholine (**a**).

Table S5: Palladium-catalyzed aminocarbonylation of iodoheteroaromatic substrates (**15-19**) with morpholine (**a**)

II. MS data set of the products

Table S6: Palladium-catalyzed aminocarbonylation of iodobenzene (**1**) with different primary and secondary amines (**a**-**h**).

Table S7: Palladium-catalyzed aminocarbonylation of *para*-substituted iodobenzene derivatives (**2-9**) with morpholine (**a**).

Table S8: Palladium-catalyzed aminocarbonylation of substituted iodobenzenes (**10-14**) with morpholine (**a**).

Table S9: Palladium-catalyzed aminocarbonylation of iodoheteroaromatic substrates (**15-19**) with morpholine (**a**)

Table S1: Optimization study of the aminocarbonylation of iodobenzene (**1**) with morpholine (**a**)[a)]

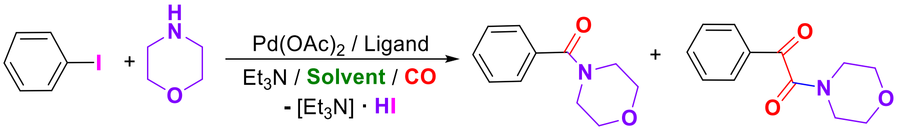

Entry	Solvent	Ligand	Temp.	R. Time	p_{CO}	Conv.[b)]	Ratio of the carbonylated products[b)]	
			[°C]	[h]	[bar]	[%]	Amide	Ketoamide
1	MetLev	PPh_3	50	6	1	29	59	41
2	MetLev	PPh_3	50	24	1	63	65	35
3	MetLev	PPh_3	70	6	1	62	88	12
4	MetLev	PPh_3	70	24	1	86	89	11
5	MetLev	PPh_3	50	6	40	63	5	95
6	MetLev	PPh_3	50	24	40	79	5	95
7	MetLev	XantPhos	50	6	1	99	100	0
8	MetLev	XantPhos	50	24	1	100	100	0
9	EtLev	PPh_3	50	6	1	46	68	32
10	EtLev	PPh_3	50	24	1	76	75	25
11	EtLev	PPh_3	70	6	1	69	91	9
12	EtLev	PPh_3	70	24	1	90	93	7
13	EtLev	PPh_3	50	6	35	78	8	92
14	EtLev	PPh_3	50	24	35	90	7	93
15	EtLev	XantPhos	50	6	1	98	100	0
16	EtLev	XantPhos	50	24	1	100	100	0
17	2-MeTHF	PPh_3	50	6	1	9	84	16
18	2-MeTHF	PPh_3	50	24	1	11	100	0
19	2-MeTHF	PPh_3	70	6	1	20	82	18
20	2-MeTHF	PPh_3	70	24	1	66	96	4
21	2-MeTHF	PPh_3	50	6	40	31	14	86
22	2-MeTHF	PPh_3	50	24	40	35	14	86
23	2-MeTHF	XantPhos	50	6	1	87	100	0
24	2-MeTHF	XantPhos	50	24	1	100	100	0

a) Reaction conditions: 0.5 mmol of iodobenzene, 0.75 mmol of morpholine, 0.0125 mmol of $Pd(OAc)_2$, 0.025 mmol of PPh_3 or 0.0125 mmol of XantPhos, 0.25 mL of triethylamine and 5 mL of solvent under CO atmosphere.
b)The conversion and the ratio of the carbonylated products were determined by GC using dodecane as an internal standard.

Table S2: Palladium-catalyzed aminocarbonylation of iodobenzene (**1**) with different amines (**b-h**) [a)]

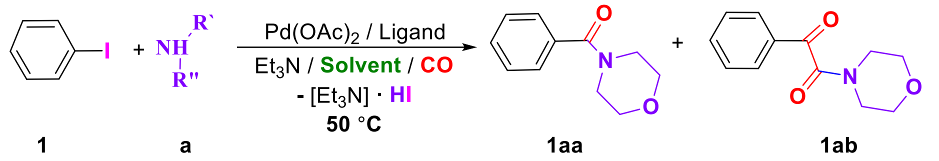

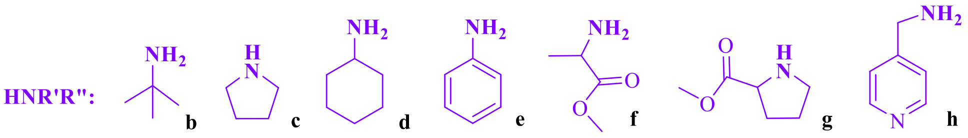

Entry	Amines	R. Time [h]	p_{CO} [bar]	Conv [%]	Conv [%]	Conv [%]
1	*t*BuNH$_2$ (**b**)	6	1	92	99	35
2	*t*BuNH$_2$ (**b**)	24	1	100	100	86
3	Pyrrolidine (**c**)	6	1	100	100	100
4	Pyrrolidine (**c**)	24	1	100	100	100
5	Cyclohexylamine (**d**)	6	1	100	100	100
6	Cyclohexylamine (**d**)	24	1	100	100	100
7	Aniline (**e**)	6	1	46	70	5
8	Aniline (**e**)	24	1	100	96	22
9	AlaOMe (**f**)	6	1	66	87	9
10	AlaOMe (**f**)	24	1	100	100	47
11	ProOMe (**g**)	6	1	84	90	6
12	ProOMe (**g**)	24	1	100	100	26
13	4-picolyamine (**h**)	6	1	66	95	100
14	4-picolyamine (**h**)	24	1	100	100	100

a) Reaction conditions: 0.5 mmol of iodobenzene, amine nucleophile (1.5 mmol of tert-butylamine, 0.75 mmol of pyrrolidine, 0.75 mmol of cyclohexylamine, 1.0 mmol of aniline, 0.55 mmol of amino acid methyl esters (AlaOMe, ProOMe), 0.75 mmol of 4-picolylamine), 0.0125 mmol of Pd(OAc)$_2$, 0.0125 mmol of XantPhos, 0.25 mL of triethylamine and 5 mL of solvent at 50 ºC under 1 bar of CO.
b)The conversion was determined by GC using dodecane as an internal standard.

Table S3: Palladium-catalyzed aminocarbonylation of *para*-substituted iodobenzene derivatives (**2-9**)[a)]

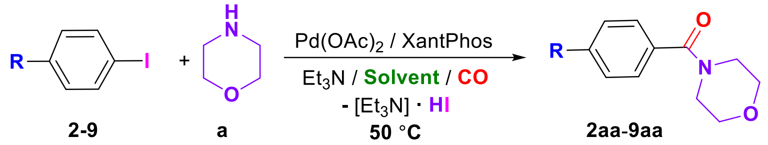

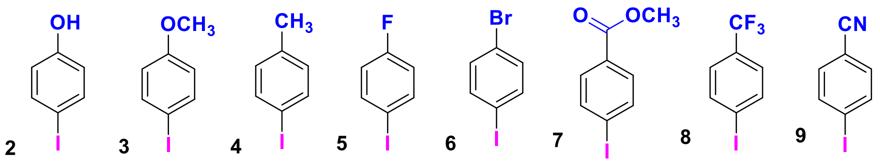

Entry	Substrate	R. Time [h]	p_{CO} [bar]	Conv. [%]	Conv. [%]	Conv. [%]
1	4-iodophenol (**2**)	2	1	96	84	22
2	4-iodophenol (**2**)	6	1	100	100	68
3	4-iodophenol (**2**)	24	1	-	-	100
4	4-iodoanisole (**3**)	2	1	76	46	10
5	4-iodoanisole (**3**)	6	1	100	92	35
6	4-iodoanisole (**3**)	24	1	-	-	94
7	4-iodotoluene (**4**)	2	1	89	67	68
8	4-iodotoluene (**4**)	6	1	99	98	100
9	1-fluoro-4-iodobenzene (**5**)	2	1	48	59	15
10	1-fluoro-4-iodobenzene (**5**)	6	1	98	91	44
11	1-fluoro-4-iodobenzene (**5**)	24	1	-	-	100
12	1-bromo-4-iodobenzene (**6**)	2	1	57	46	11
13	1-bromo-4-iodobenzene (**6**)	6	1	98	100	32
14	1-bromo-4-iodobenzene (**6**)	24	1	-	-	92
15	methyl 4-iodo-benzoate (**7**)	2	1	82	66	45
16	methyl 4-iodo-benzoate (**7**)	6	1	100	99	100
17	4-iodobenzotrifluoride (**8**)	2	1	58	67	11
18	4-iodobenzotrifluoride (**8**)	6	1	100	97	24
19	4-iodobenzotrifluoride (**8**)	24	1	-	-	78
20	4-iodobenzonitrile (**9**)	2	1	67	55	13
21	4-iodobenzonitrile (**9**)	6	1	100	100	39
22	4-iodobenzonitrile (**9**)	24	1	-	-	100

a) Reaction conditions: 0.5 mmol of *para*-substituted iodobenzene, 0.75 mmol of morpholine, 0.0125 mmol of Pd(OAc)$_2$, 0.0125 mmol of XantPhos, 0.25 mL of triethylamine and 5 mL of solvent under 1 bar of CO

b) The conversion was determined by GC using dodecane as an internal standard.

Table S4: Palladium-catalyzed aminocarbonylation of substituted iodobenzenes (**10-14**) with morpholine (**a**) [a)]

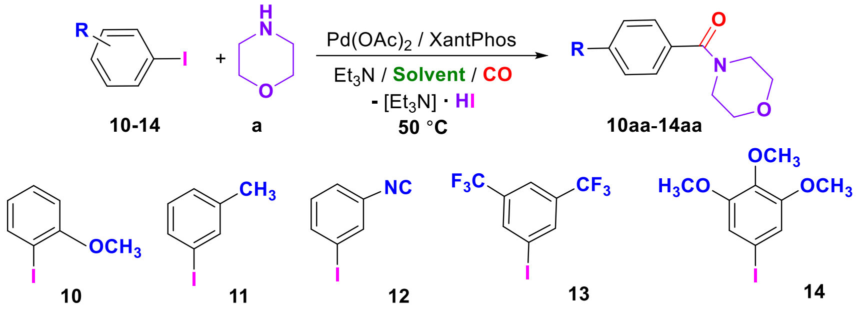

Entry	Substrate	R. Time [h]	pco [bar]	Conv. [%]	Conv. [%]	Conv. [%]
1	2-iodoanisole (**10**)	2	1	36	31	17
2	2-iodoanisole (**10**)	6	1	57	60	33
3	2-iodoanisole (**10**)	23	1	83	80	58
4	2-iodoanisole (**10**)	48	1	84	80	63
5	3-iodotoluene (**11**)	2	1	55	49	9
6	3-iodotoluene (**11**)	6	1	96	94	26
7	3-iodotoluene (**11**)	24	1	-	100	97
8	3-iodobenzonitrile (**12**)	2	1	33	36	6
9	3-iodobenzonitrile (**12**)	6	1	71	84	16
10	3-iodobenzonitrile (**12**)	23	1	100	99	60
11	3-iodobenzonitrile (**12**)	48	1	-	-	86
12	1-iodo-3,5-bis(trifluoromethyl)benzene (**13**)	2	1	16	11	3
13	1-iodo-3,5-bis(trifluoromethyl)benzene (**13**)	6	1	37	31	6
14	1-iodo-3,5-bis(trifluoromethyl)benzene (**13**)	24	1	93	88	16
15	5-iodo-1,2,3-trimethoxybenzene (**14**)	2	1	17	14	7
16	5-iodo-1,2,3-trimethoxybenzene (**14**)	6	1	46	50	21
17	5-iodo-1,2,3-trimethoxybenzene (**14**)	23	1	100	100	65
18	5-iodo-1,2,3-trimethoxybenzene (**14**)	48	1	-	-	100

a) Reaction conditions: 0.5 mmol of substrate, 0.75 mmol of morpholine, 0.0125 mmol of $Pd(OAc)_2$, 0.0125 mmol of XantPhos, 0.25 mL of triethylamine and 5 mL of solvent under 1 bar of CO.

b) The conversion was determined by GC using dodecane as an internal standard.

Table S5: Palladium-catalyzed aminocarbonylation of iodoheteroaromatic substrates (**15-19**) with morpholine (**a**)[a)]

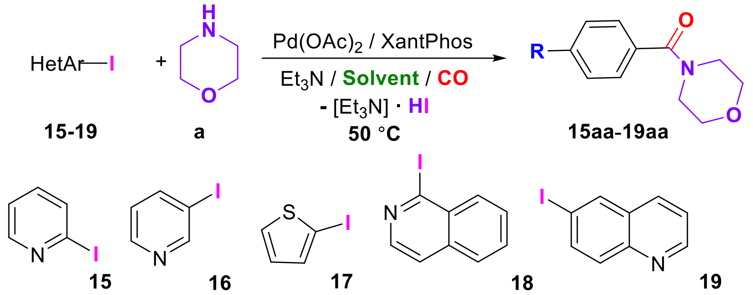

Entry	Substrate	R. Time [h]	p_{CO} [bar]	Conv. [%]	Conv. [%]	Conv. [%]
1	2-iodopyridine (**15**)	2	1	100	100	20
2	2-iodopyridine (**15**)	6	1	-	-	82
3	2-iodopyridine (**15**)	24	1	-	-	100
4	3-iodopyridine (**16**)	2	1	43	47	11
5	3-iodopyridine (**16**)	6	1	88	92	36
6	3-iodopyridine (**16**)	23	1	100	100	98
7	2-iodothiophene (**17**)	2	1	60	82	5
8	2-iodothiophene (**17**)	6	1	96	97	7
9	2-iodothiophene (**17**)	24	1	-	-	27
10	1-iodoisoquinoline (**18**)	2	1	100	100	9
11	1-iodoisoquinoline (**18**)	6	1	-	-	25
12	1-iodoisoquinoline (**18**)	24	1	-	-	100
13	6-iodoquinoline (**19**)	2	1	80	83	7
14	6-iodoquinoline (**19**)	6	1	100	100	14
15	6-iodoquinoline (**19**)	24	1	-	100	67

a) Reaction conditions: 0.5 mmol of substrate, 0.75 mmol of morpholine, 0.0125 mmol of $Pd(OAc)_2$, 0.0125 mmol of XantPhos, 0.25 mL of triethylamine and 5 mL of solvent under 1 bar of CO.

b) The conversion was determined by GC using dodecane as an internal standard.

Table S6: Palladium-catalyzed aminocarbonylation of iodobenzene (**1**) with different primary and secondary amines (**a**-**h**).

Product	MS m/z (rel. int.)	
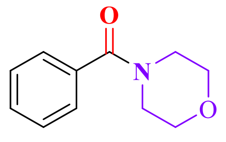 **1aa**	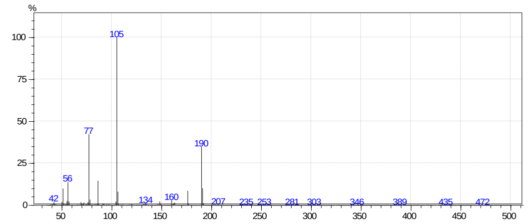	191 (10, M⁺), 190 (35), 176 (9), 105 (100), 86 (15), 77 (42), 56 (14), 51 (10)
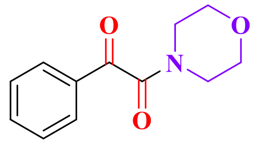 **1ab**	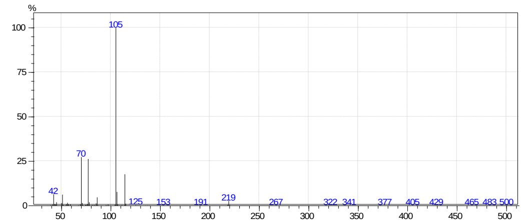	219 (3, M⁺), 114 (18), 105 (100), 86 (5), 77 (26), 70 (27), 51 (6), 42 (6)
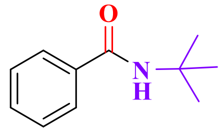 **1ba**	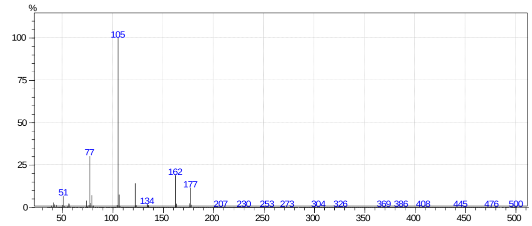	177 (12, M⁺), 162 (19), 122 (14), 105 (100), 77 (30), 73 (4), 51 (7)
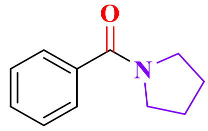 **1ca**	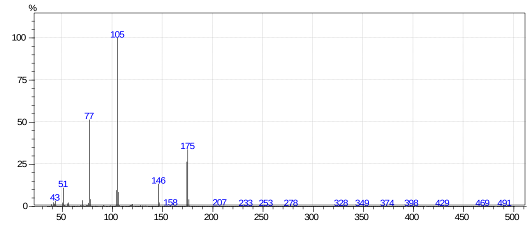	175 (34, M⁺), 174 (26), 146 (13), 105 (100), 77 (52), 70 (4), 56 (2), 51 (11), 43 (3)
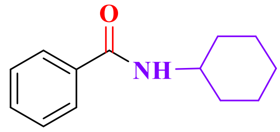 **1da**	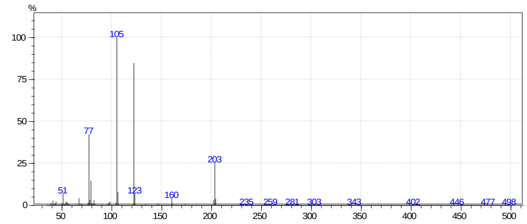	203 (25, M⁺), 160 (4), 122 (85), 105 (100), 79 (15), 77 (42), 67 (4), 51 (7), 41 (3)
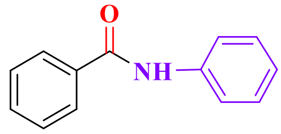 **1ea**	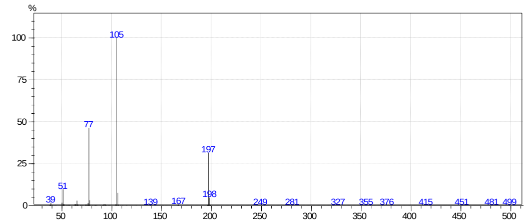	197 (31, M⁺), 106 (8), 105 (100), 77 (46), 65 (3), 51 (10)
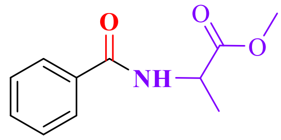 **1fa**	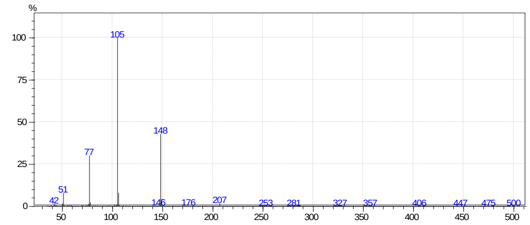	207 (1.68, M⁺), 148 (43), 106 (8), 105 (100), 77 (30), 51 (8)

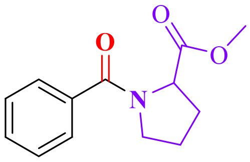 **1ga**	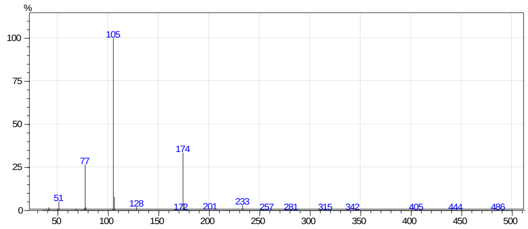	233 (3, M+), 174 (34), 128 (2), 106 (8), 105 (100), 77 (27), 51 (5), 41 (2)
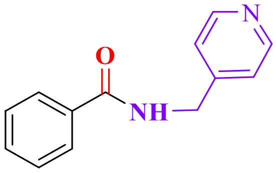 **1ha**	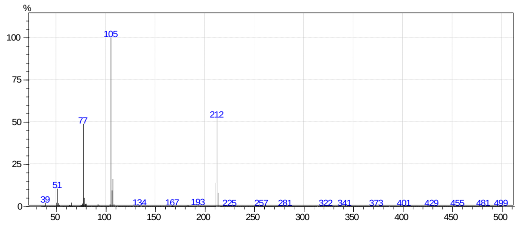	212 (53, M+), 213 (8), 211 (14), 107 (16), 106 (10), 105 (100), 77 (49), 51 (11), 39 (2)

Table S7: Palladium-catalyzed aminocarbonylation of *para*-substituted iodobenzene derivatives (**2-9**) with morpholine (**a**).

Product	MS m/z (rel. int.)	
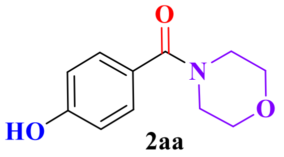 **2aa**	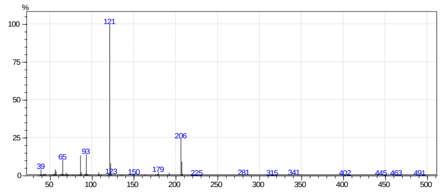	207 (9, M+), 206 (25), 192 (2), 179 (2), 122 (8), 121 (100), 93 (14), 86 (13), 65 (11), 56 (4), 39 (4)
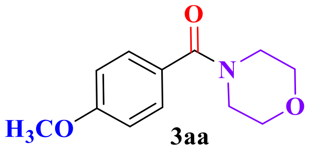 **3aa**	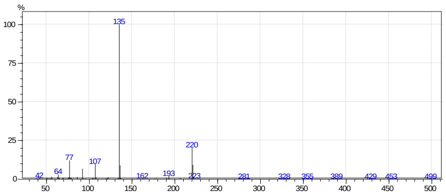	221 (9, M+), 220 (20), 193 (2), 135 (100), 107 (10), 92 (7), 77 (12), 64 (3), 56 (2)
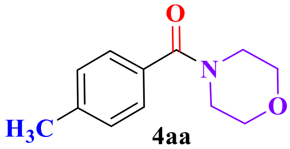 **4aa**	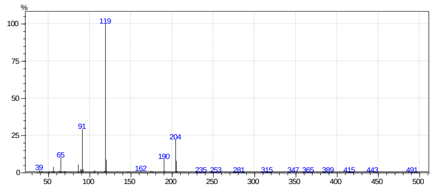	205 (8, M+), 204 (22), 190 (9), 193 (2), 120 (9), 119 (100), 91 (29), 86 (6), 65 (10), 56 (4), 39 (2)
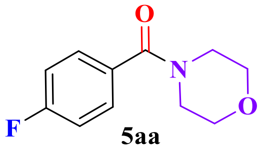 **5aa**	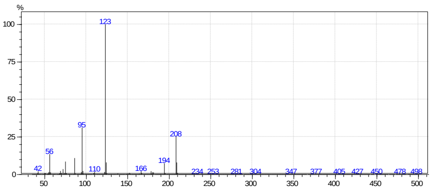	209 (8, M+), 208 (25), 194 (7), 124 (8), 123 (100), 95 (31), 86 (11), 75 (9), 56 (14), 42 (2)
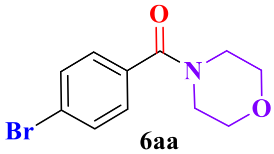 **6aa**	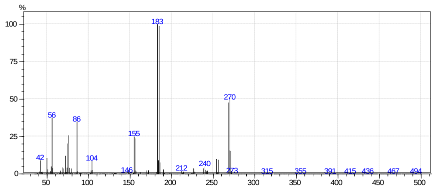	270 / 268 (49 / 48 M+), 256 / 254 (10), 185 / 183 (99 / 100), 157 / 155 (24 / 25), 104 (9), 86 (35), 76 (26), 56 (38), 42 (9)
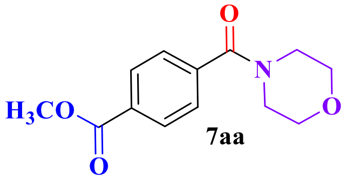 **7aa**	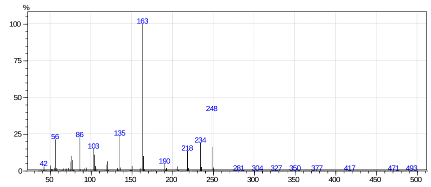	249 (16, M+), 248 (40), 234 (19), 218 (14), 190 (5), 163 (100), 135 (24), 103 (15), 86 (23), 76 (10), 56 (22), 42 (3)
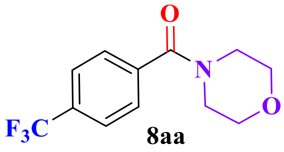 **8aa**	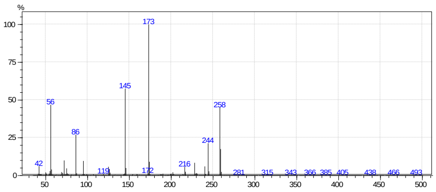	259 (17, M+), 258 (45), 244 (21), 216 (6), 173 (100), 145 (58), 125 (6), 95 (10), 86 (27), 72 (10), 56 (47), 42 (6)

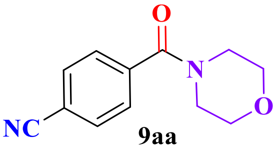 **9aa**	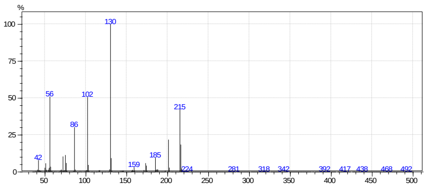	216 (18, M⁺), 215 (42), 201 (22), 185 (10), 130 (100), 102 (51), 86 (30), 75 (11), 56 (51), 42 (8)

Table S8: Palladium-catalyzed aminocarbonylation of substituted iodobenzenes (**10**-**14**) with morpholine (**a**).

Product	MS m/z (rel. int.)	
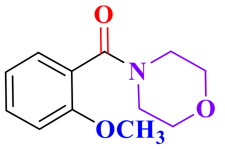 **10aa**	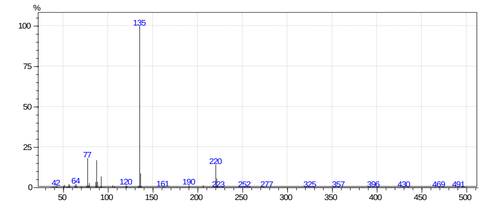	221 (6, M^+), 220 (14), 136 (9), 135 (100), 92 (7), 87 (17), 77 (18), 51 (2)
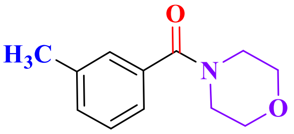 **11aa**	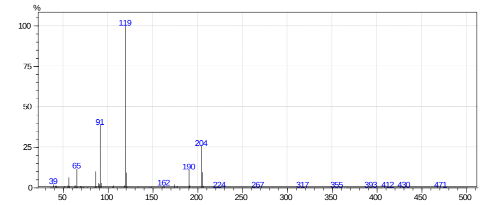	205 (10, M^+), 204 (26) 190 (11), 119 (100), 91 (39), 86 (10), 65 (12), 56 (6)
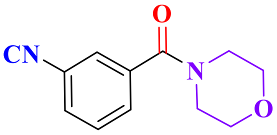 **12aa**	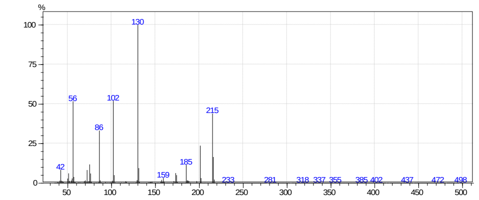	216 (17, M^+), 215 (44), 201 (24), 185 (11), 130 (100), 102 (52), 86 (33), 75 (12), 56 (52), 42 (8)
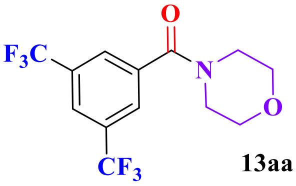 **13aa**	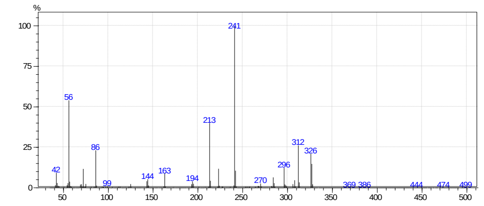	327 (15, M^+), 326 (21), 312 (26), 296 (12), 241 (100), 213 (40), 163 (9), 86 (23), 72 (12), 56 (54), 42 (9)
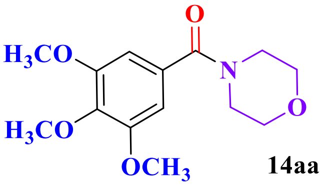 **14aa**	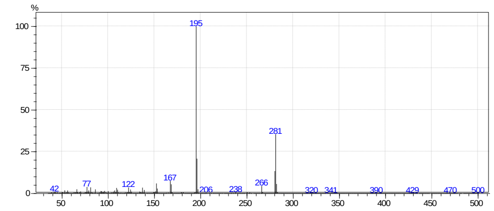	281 (35, M^+), 282 (6), 280 (13), 266 (4), 196 (21), 195 (100), 167 (7), 152 (6), 122 (4), 77 (4), 66 (3), 53 (2)

Table S9: Palladium-catalyzed aminocarbonylation of iodoheteroaromatic substrates (**15-19**) with morpholine (**a**)

Product	MS m/z (rel. int.)	
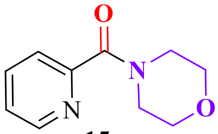 **15aa**	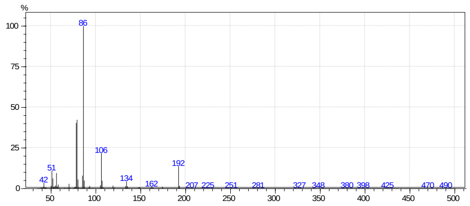	192 (14, M$^+$), 134 (4), 106 (22), 86 (100), 79 (42), 78 (40), 56 (10), 51 (11), 42 (4)
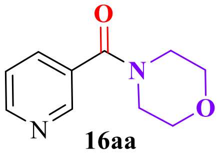 **16aa**	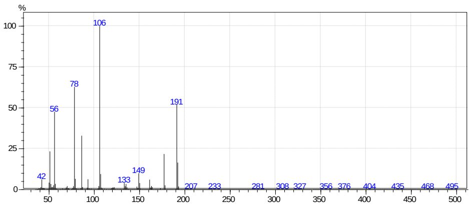	192 (16, M$^+$), 191 (52), 177 (22), 149 (10), 106 (100), 86 (33), 78 (63), 56 (48), 51 (23)
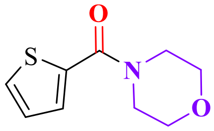 **17aa**	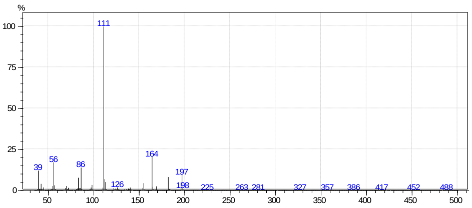	197 (9, M$^+$), 182 (8), 164 (20), 155 (4), 111 (100), 86 (14), 83 (8), 56 (17), 39 (12)
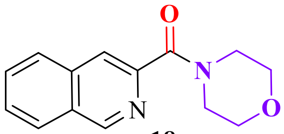 **18aa**	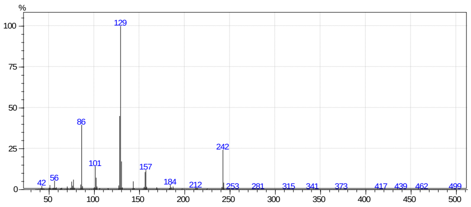	242 (24, M$^+$), 157 (12), 156 (11), 143 (5), 129 (100), 128 (45), 101 (14), 86 (39), 77 (6), 56 (5)
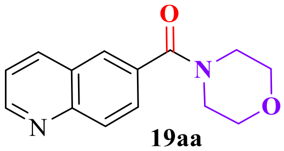 **19aa**	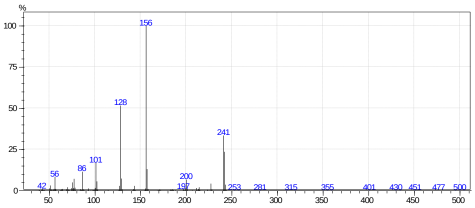	242 (24, M$^+$), 241 (33), 227 (4), 200 (7), 156 (100), 157 (13), 101 (17), 86 (12), 77 (7), 75 (5), 56 (8)
